# Supplementary figures and images for: Antigenic cancer persister cells survive direct T cell attack
Source: bioRxiv. 2025 Mar 17:2025.03.14.643359. Preprint. [Version 1] doi: 10.1101/2025.03.14.643359 (PMC11956947; doi:10.1101/2025.03.14.643359)

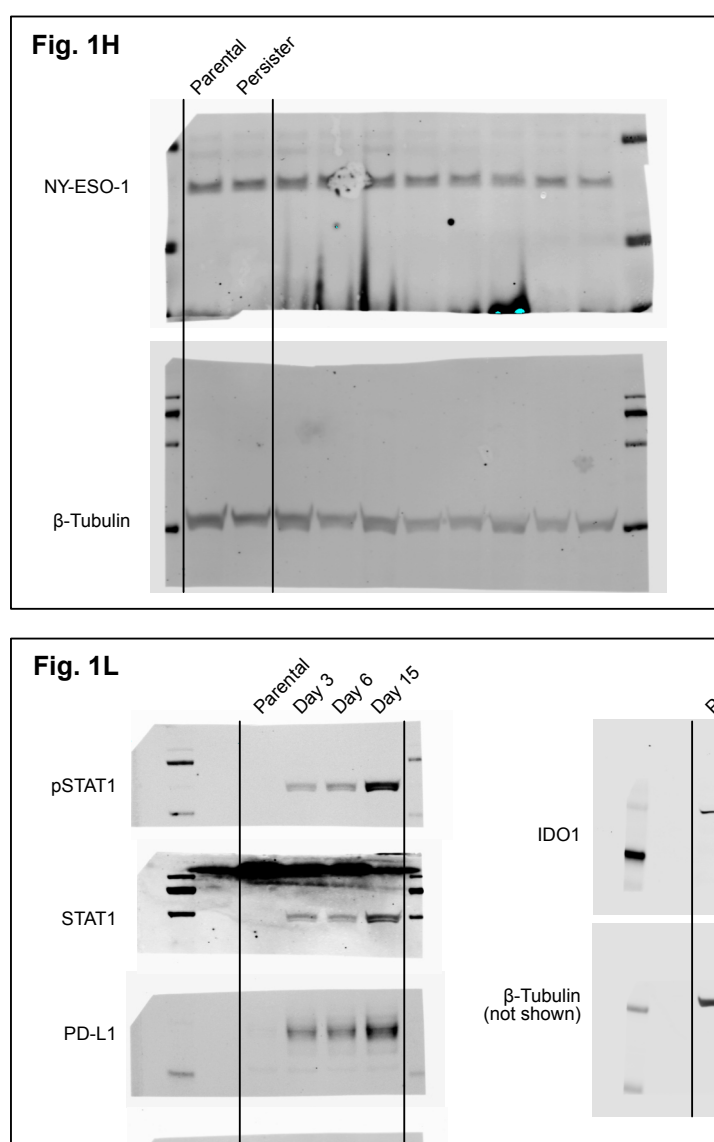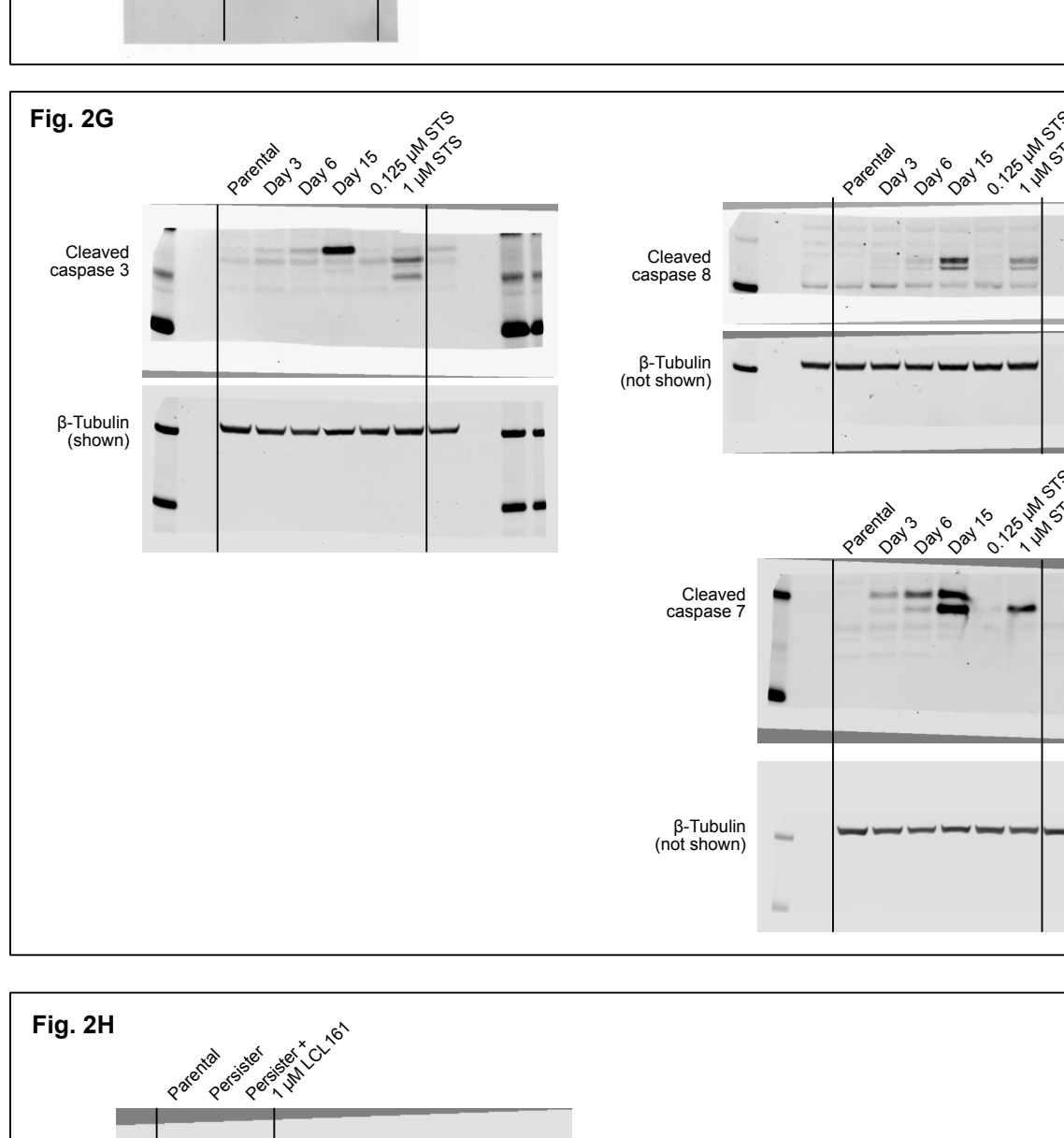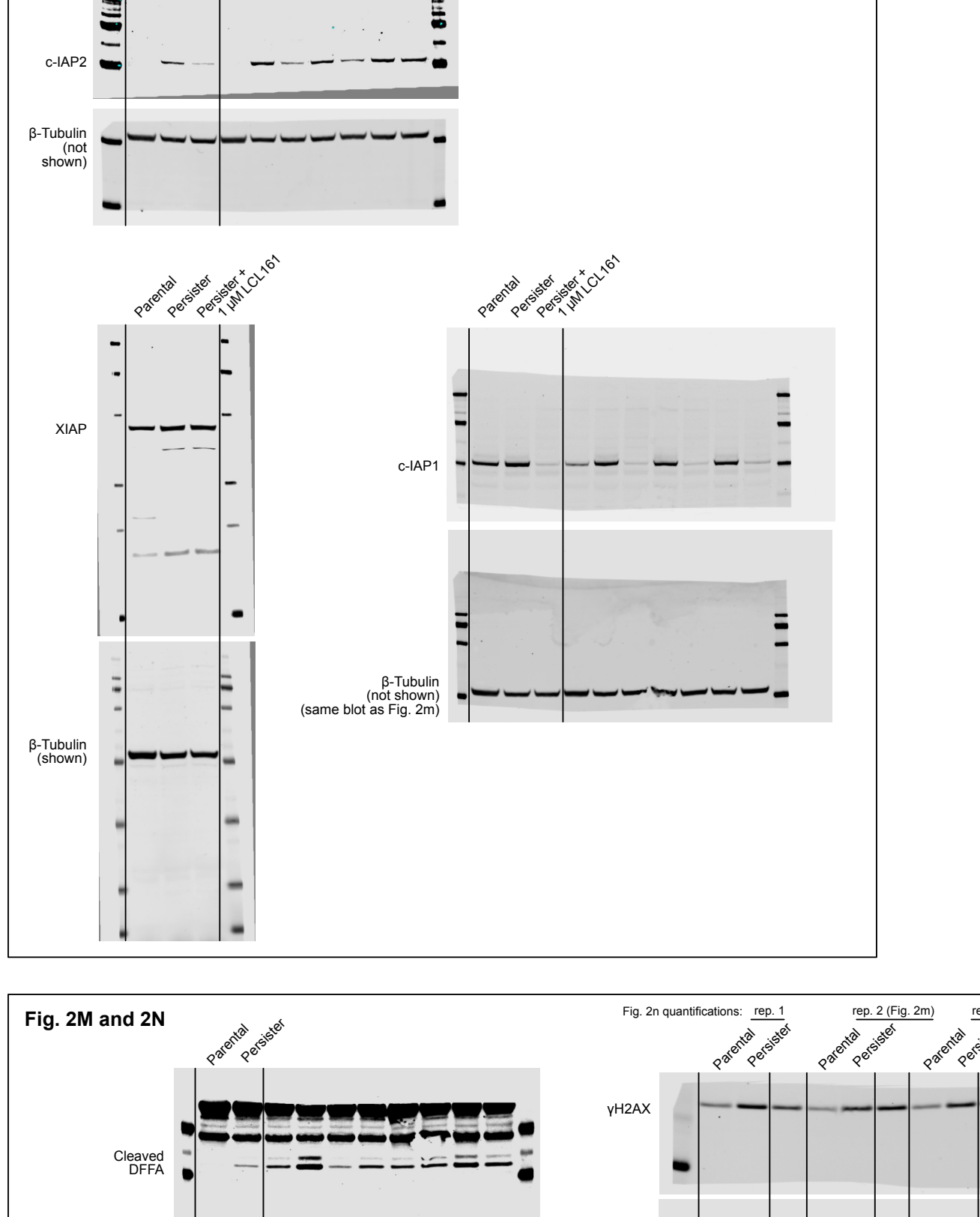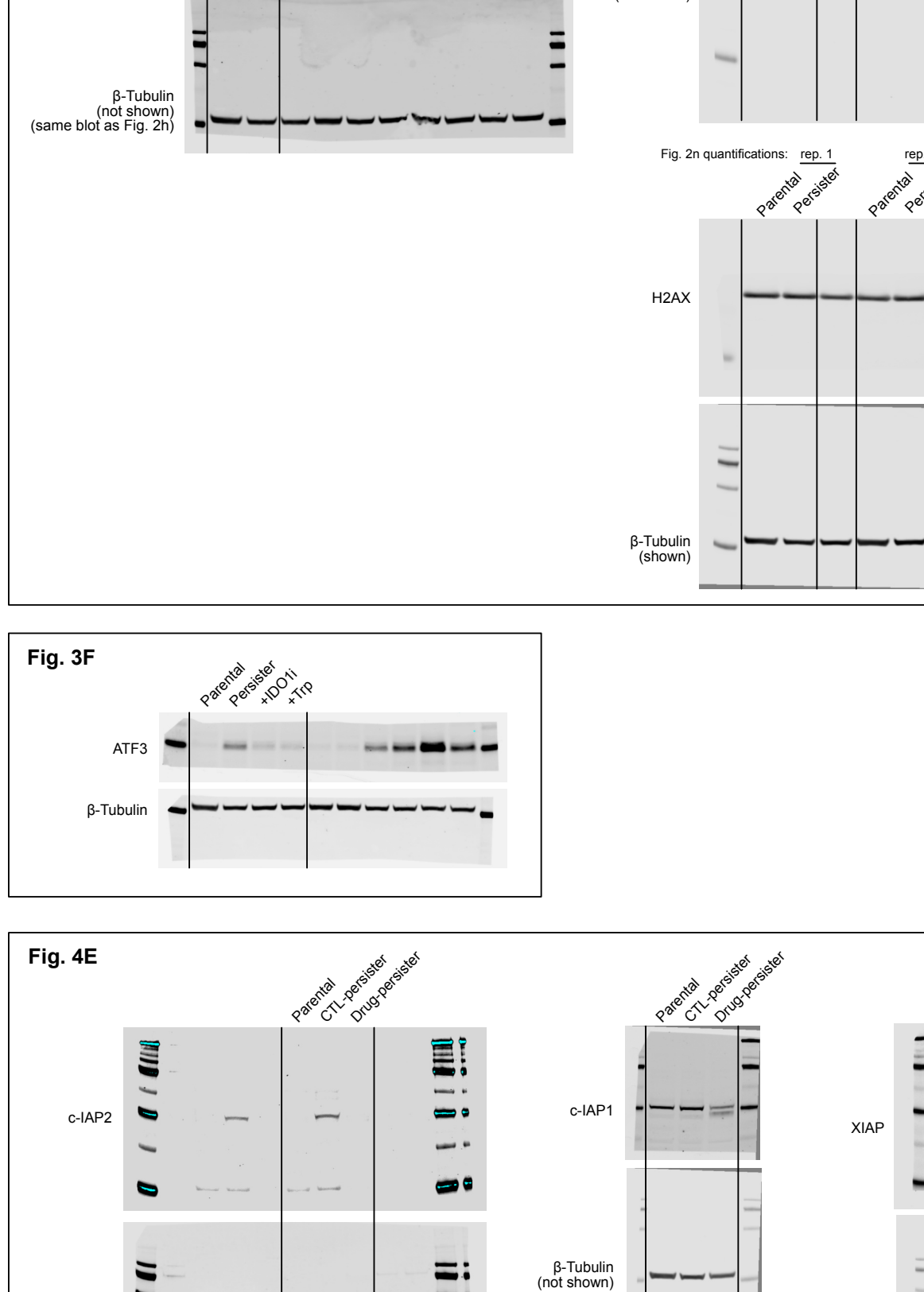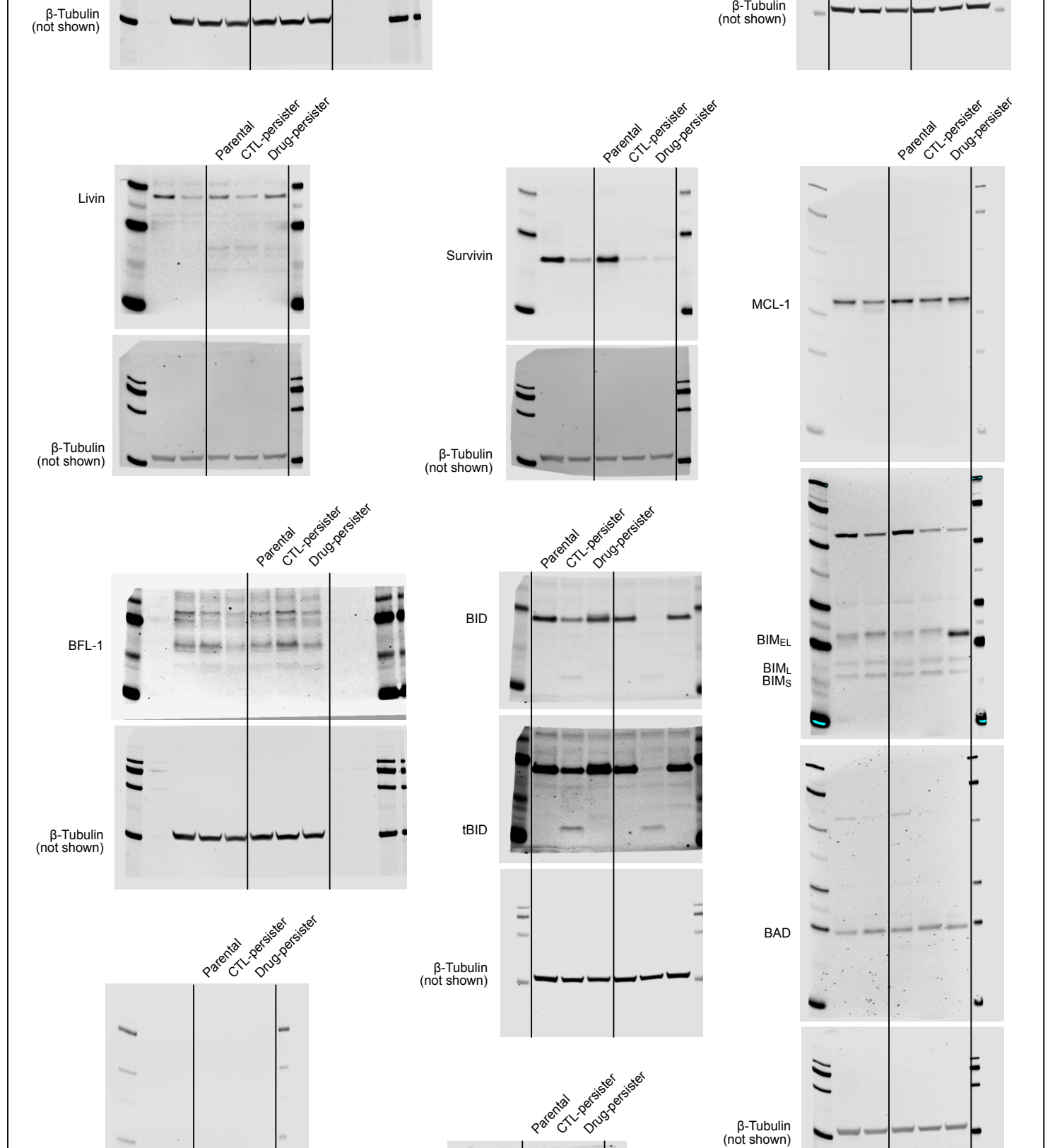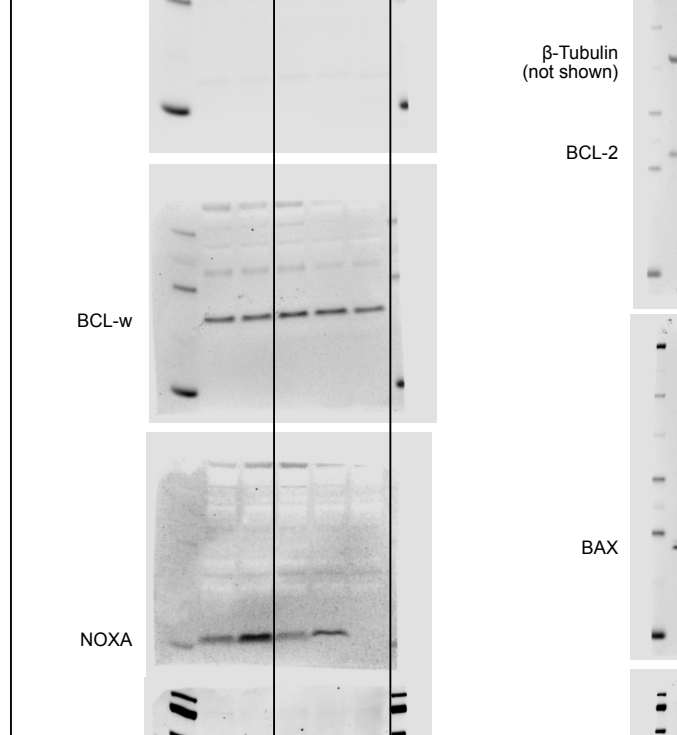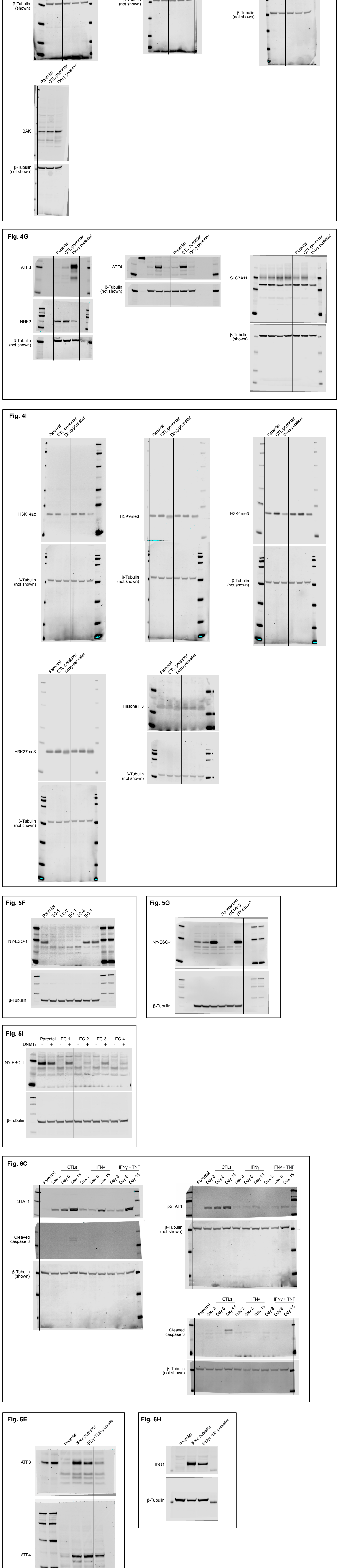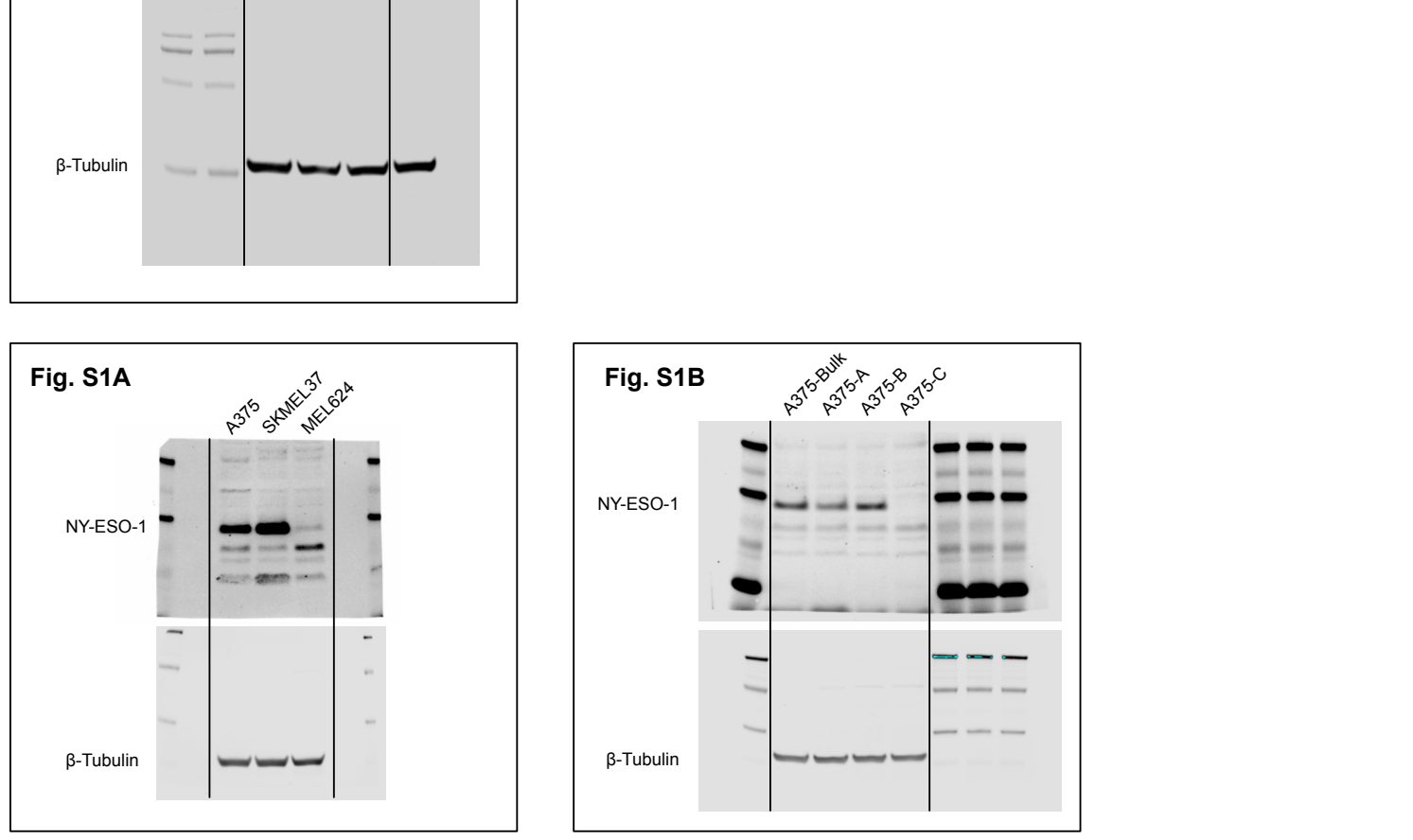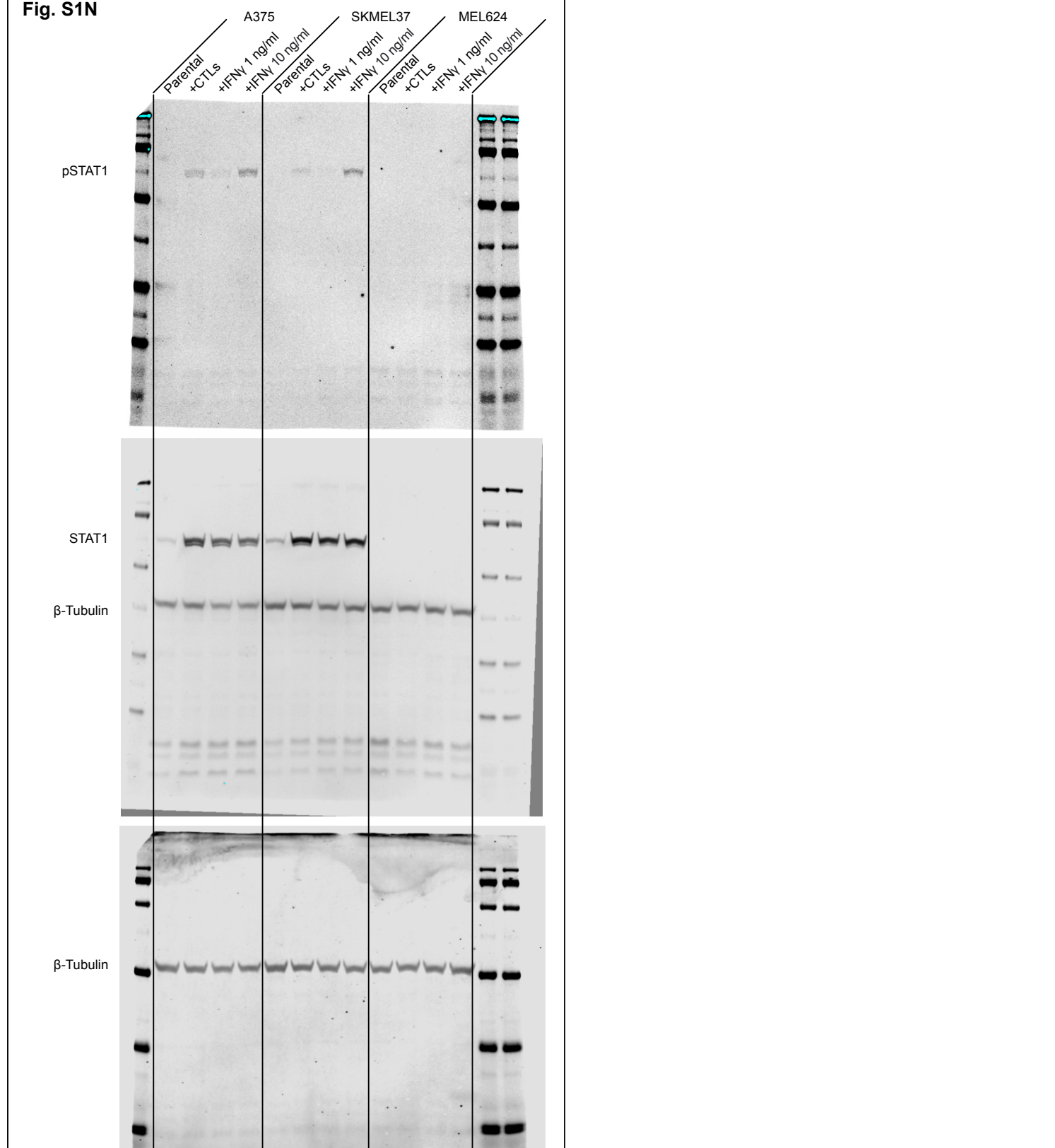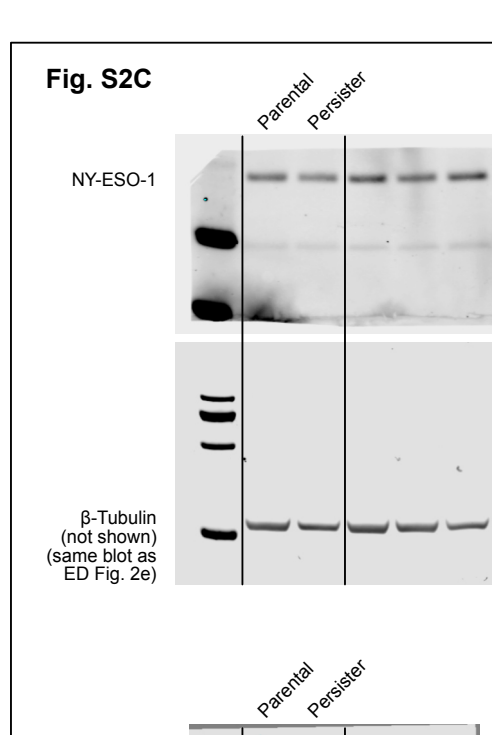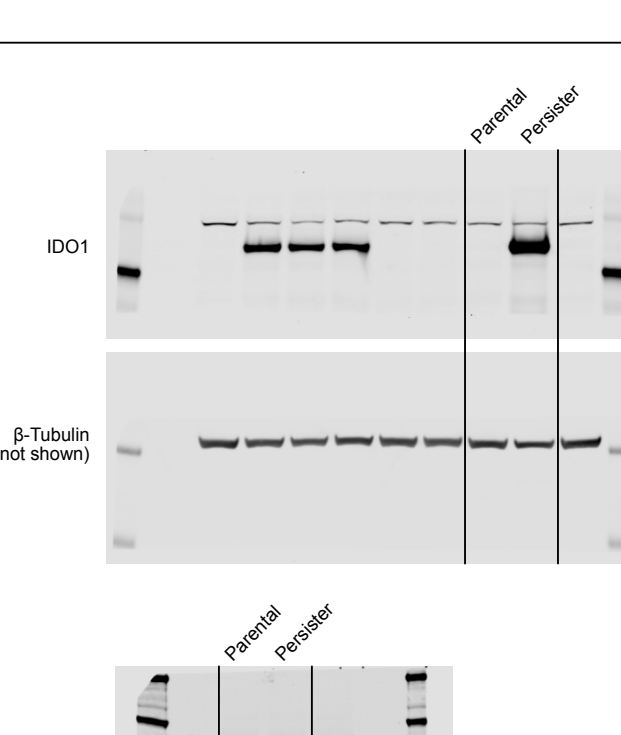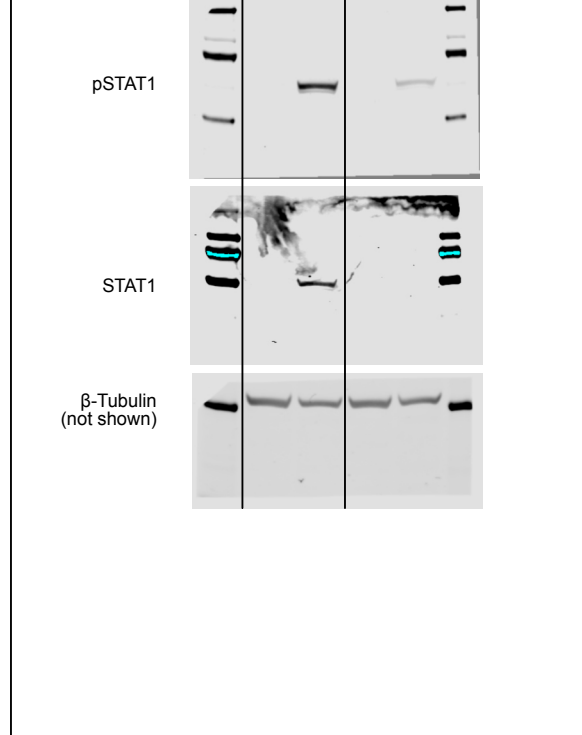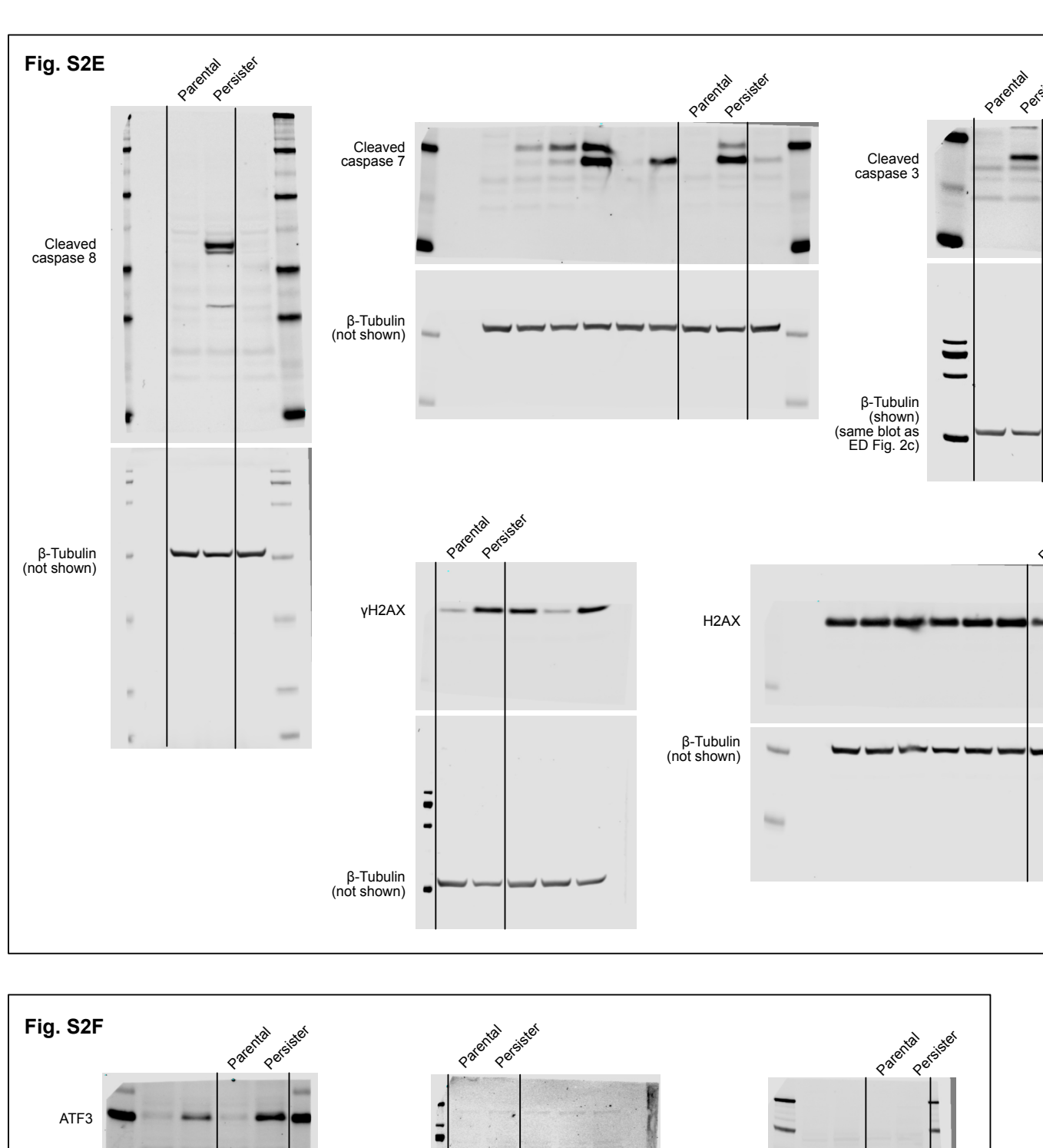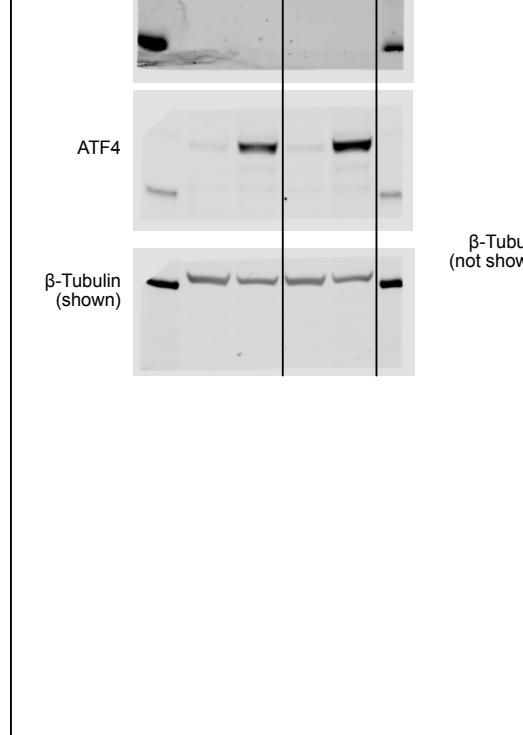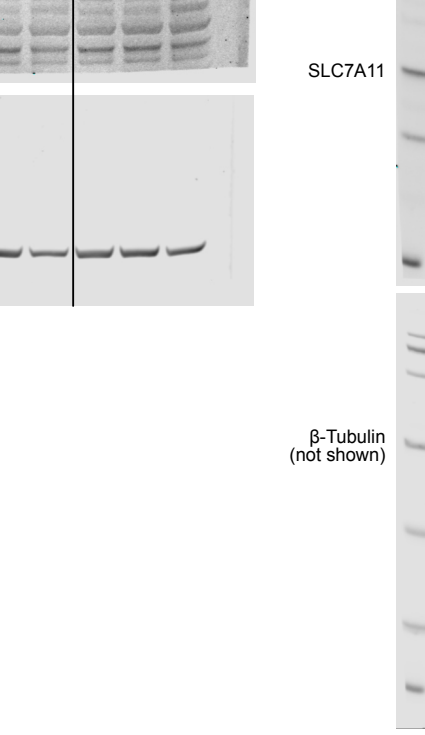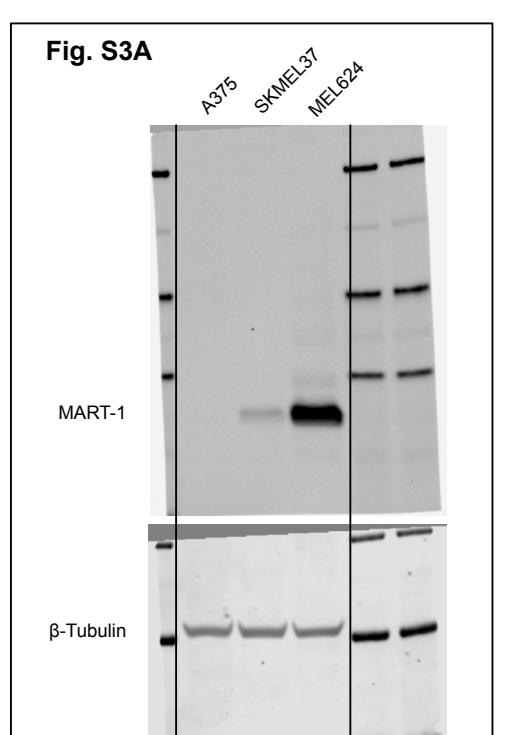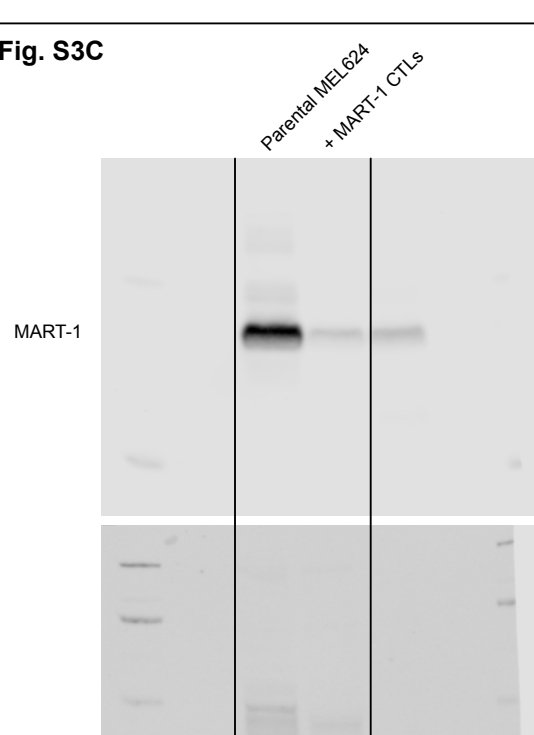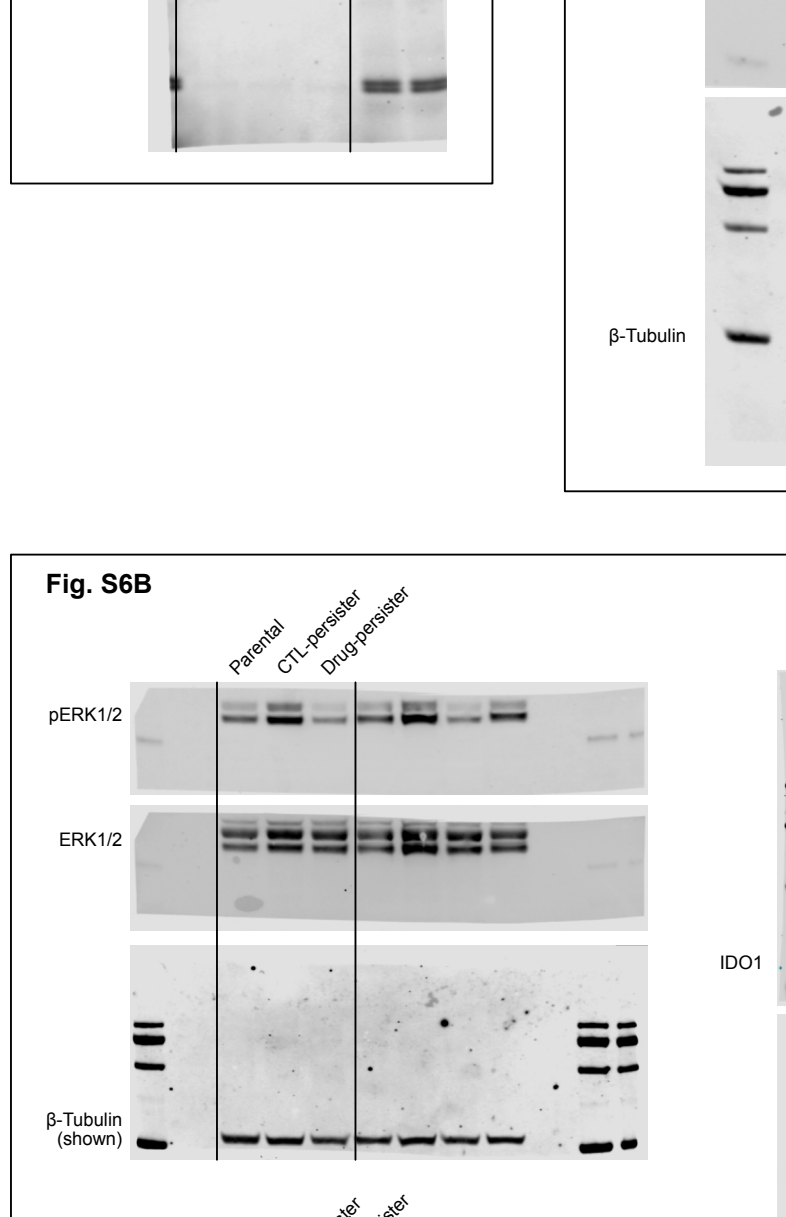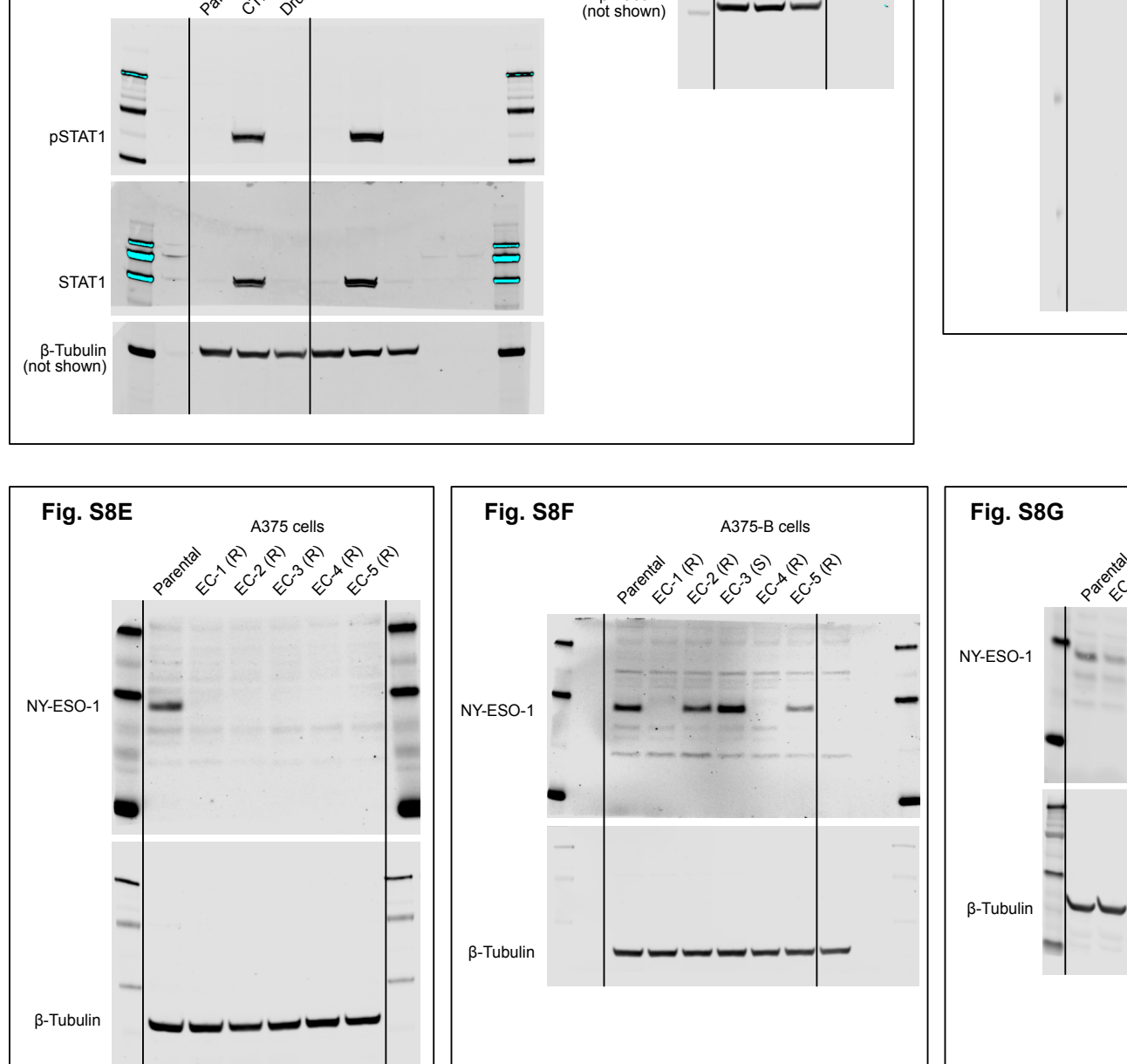

Supplement: Supplement 2 — Document S2. Figure S10 (uncropped western blot images) [file media-2.pdf]

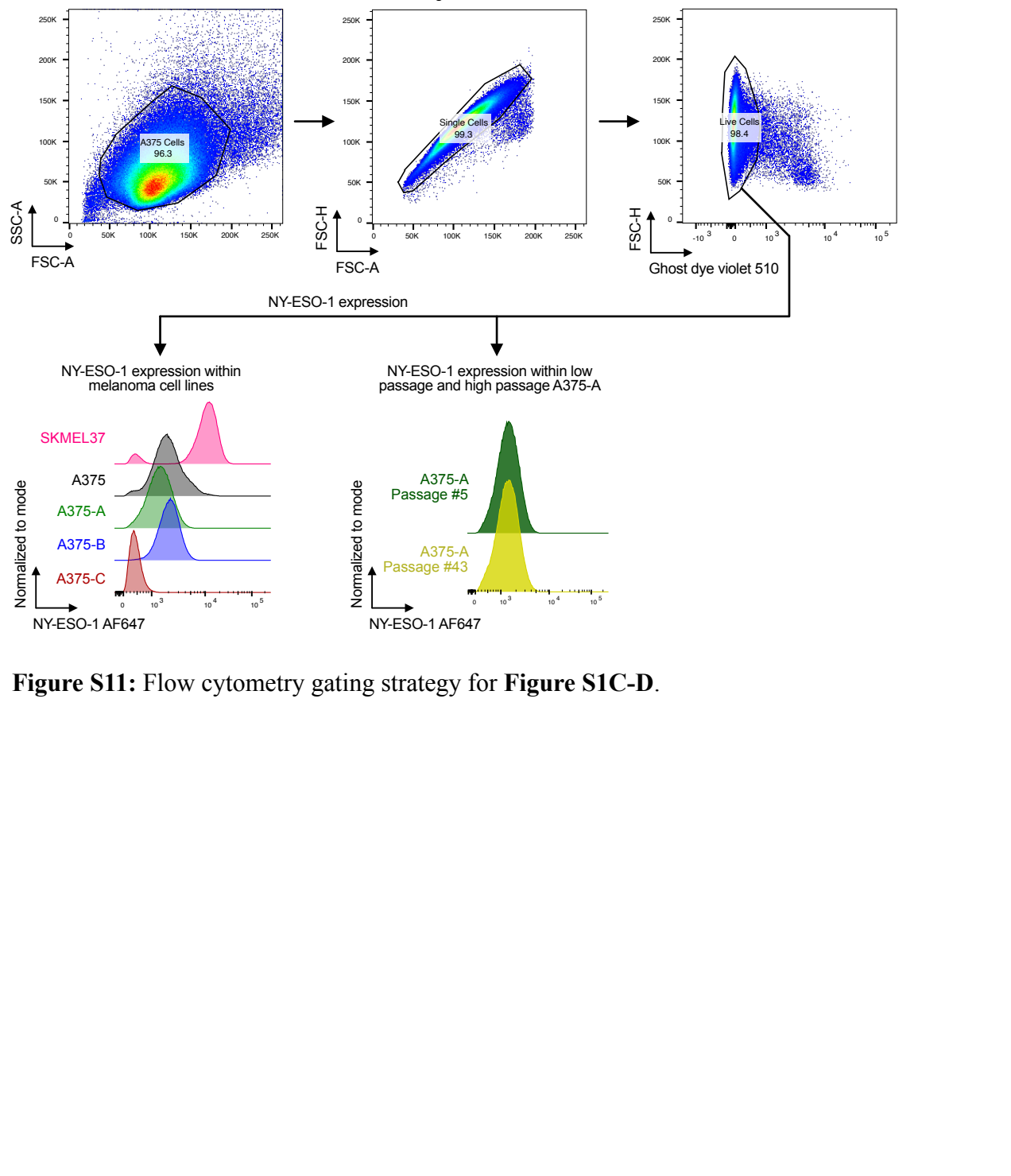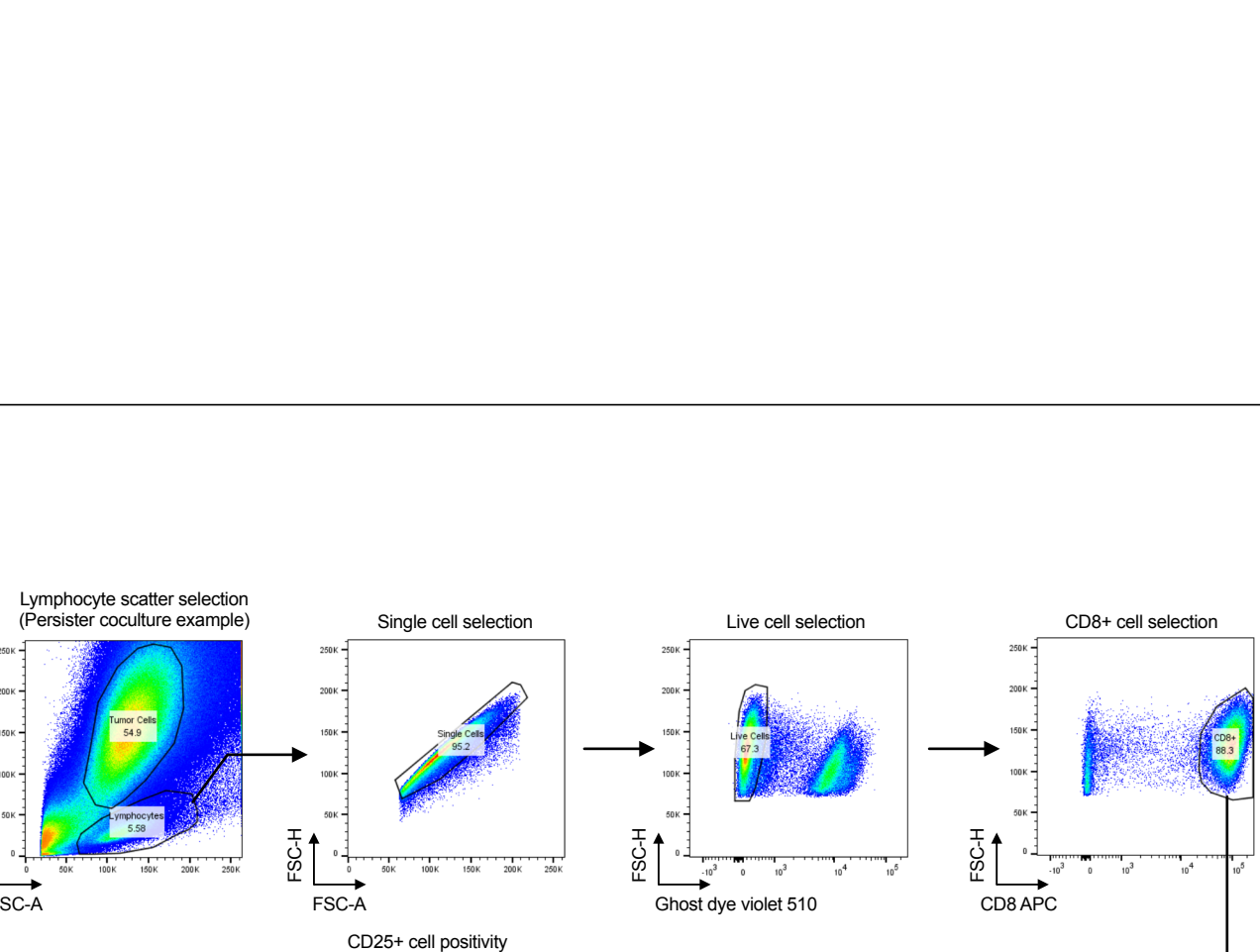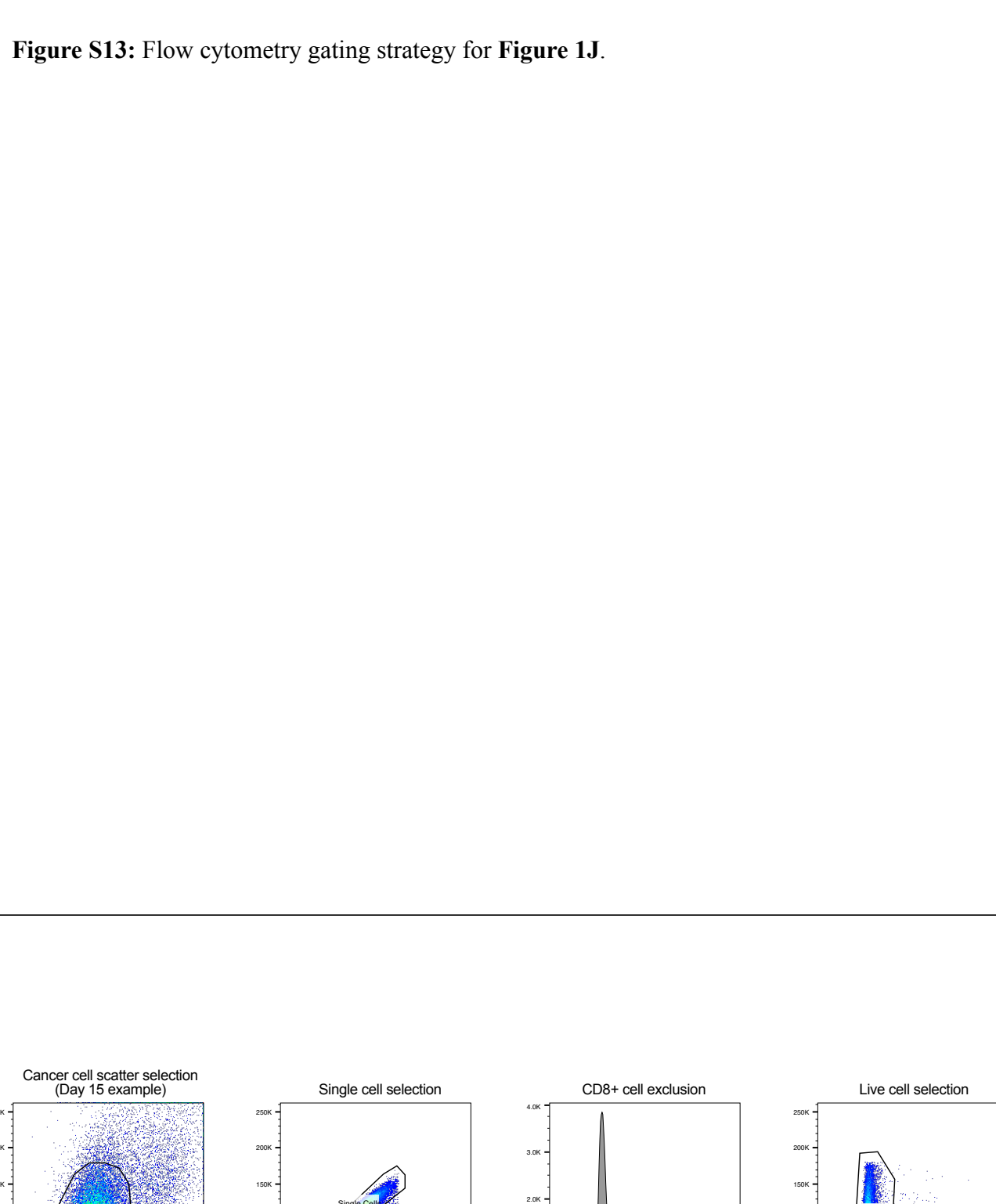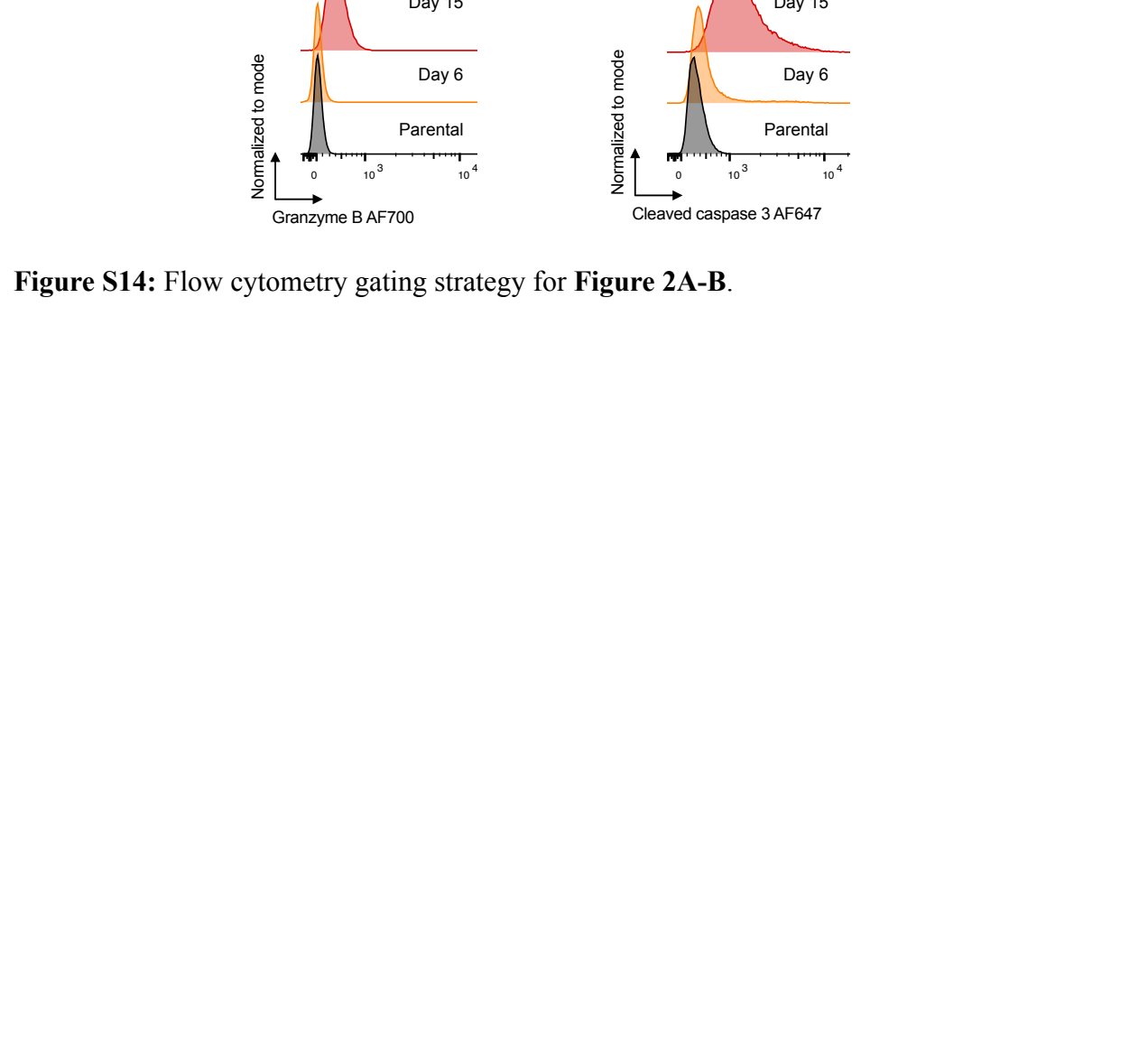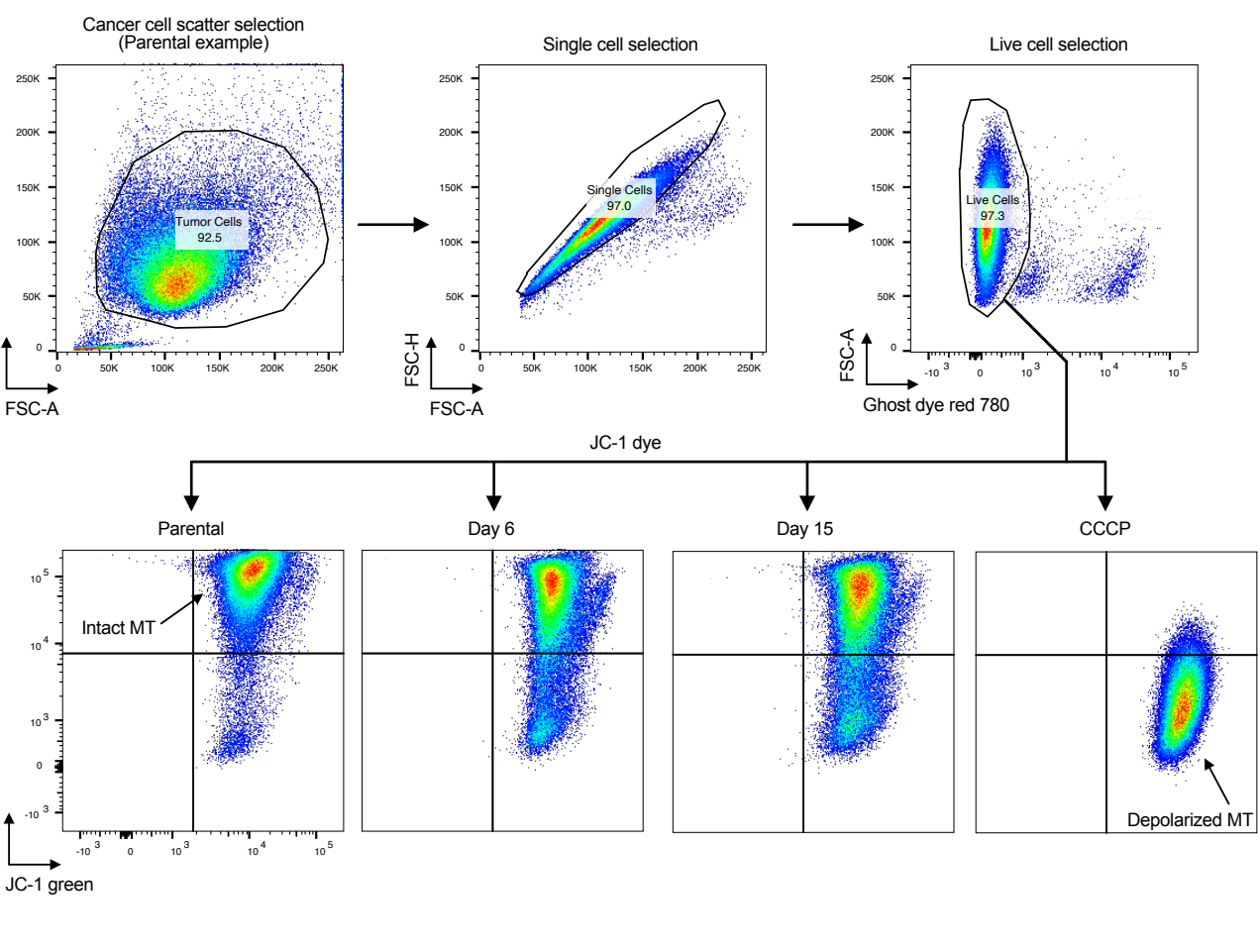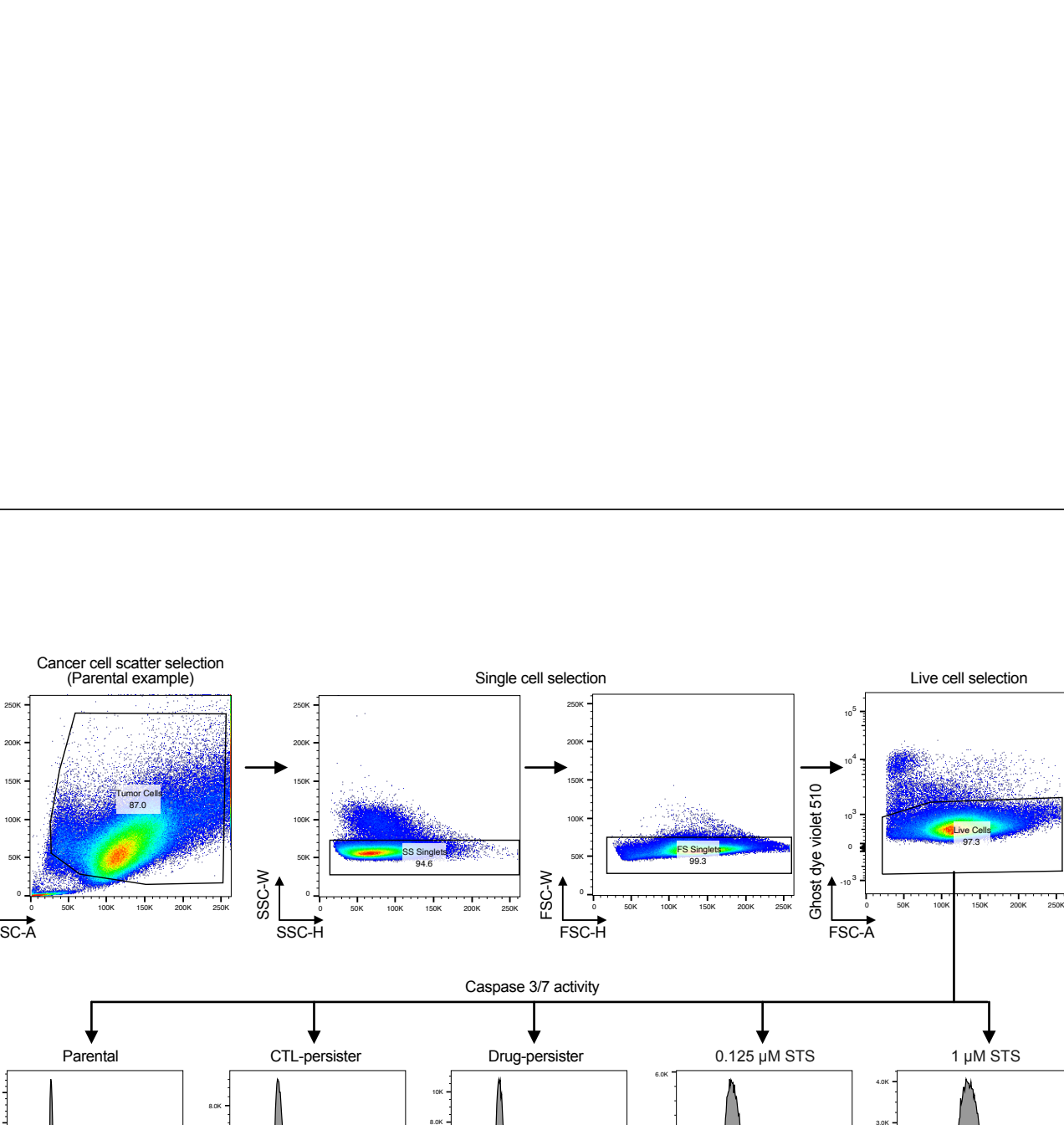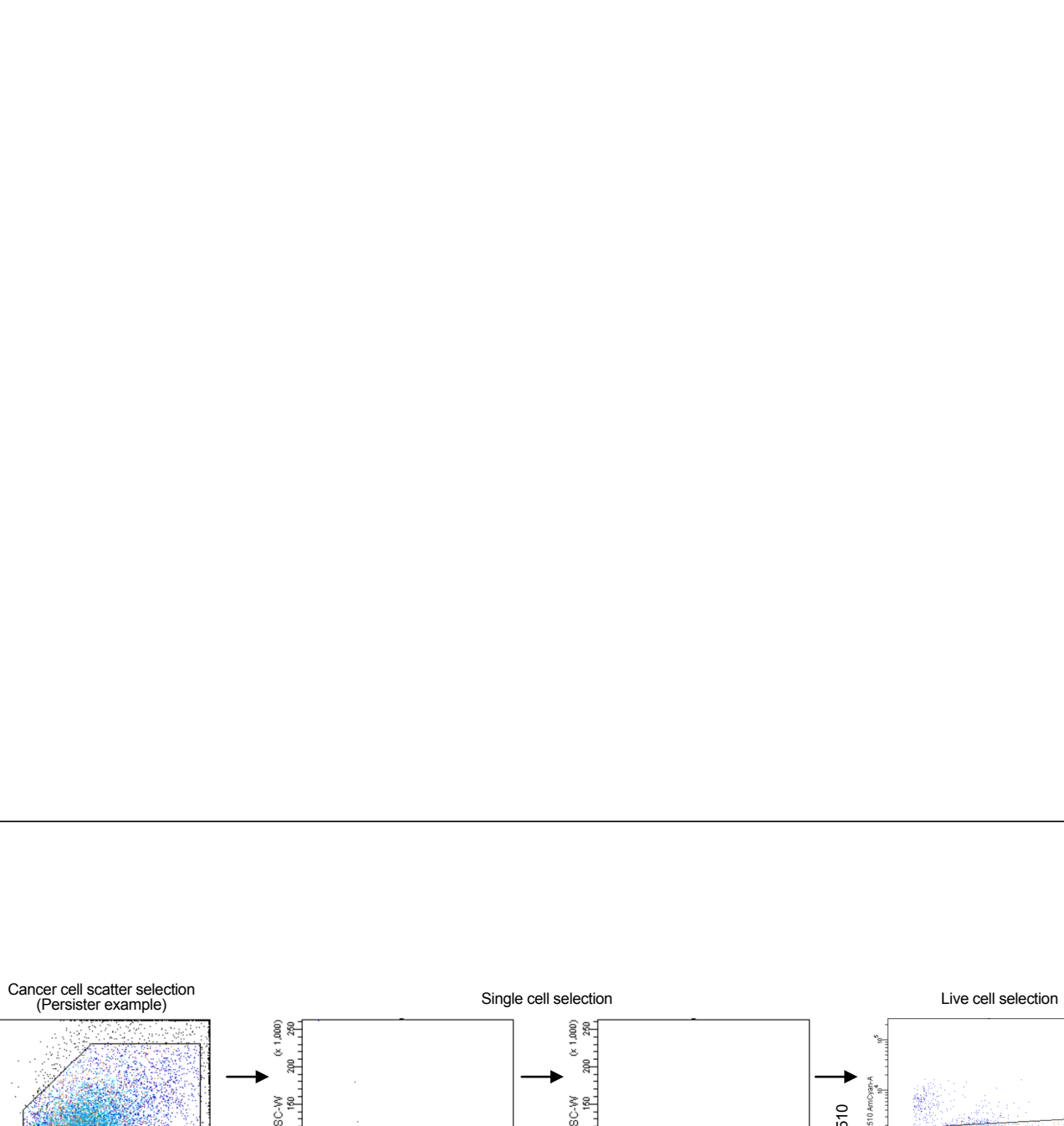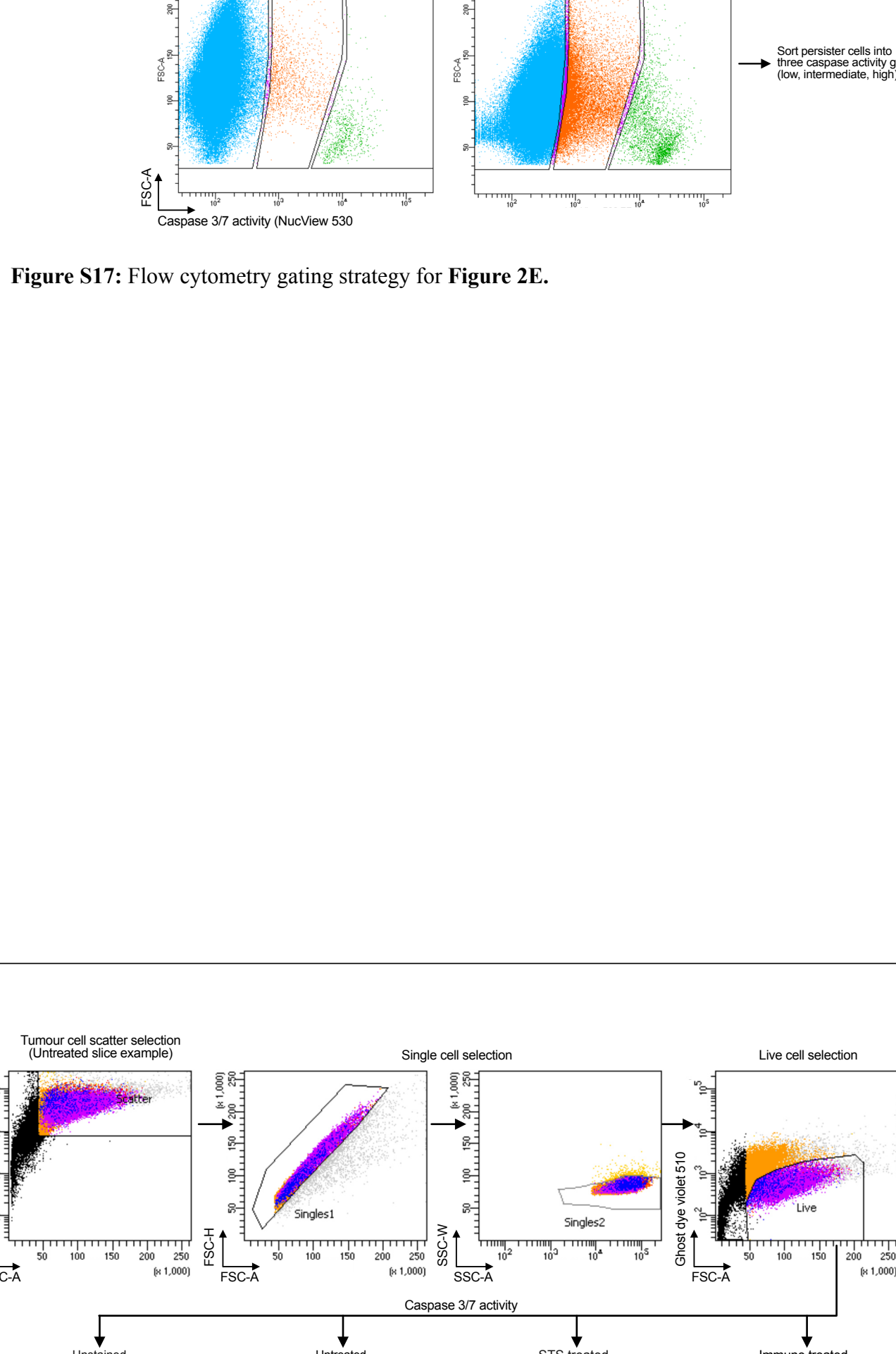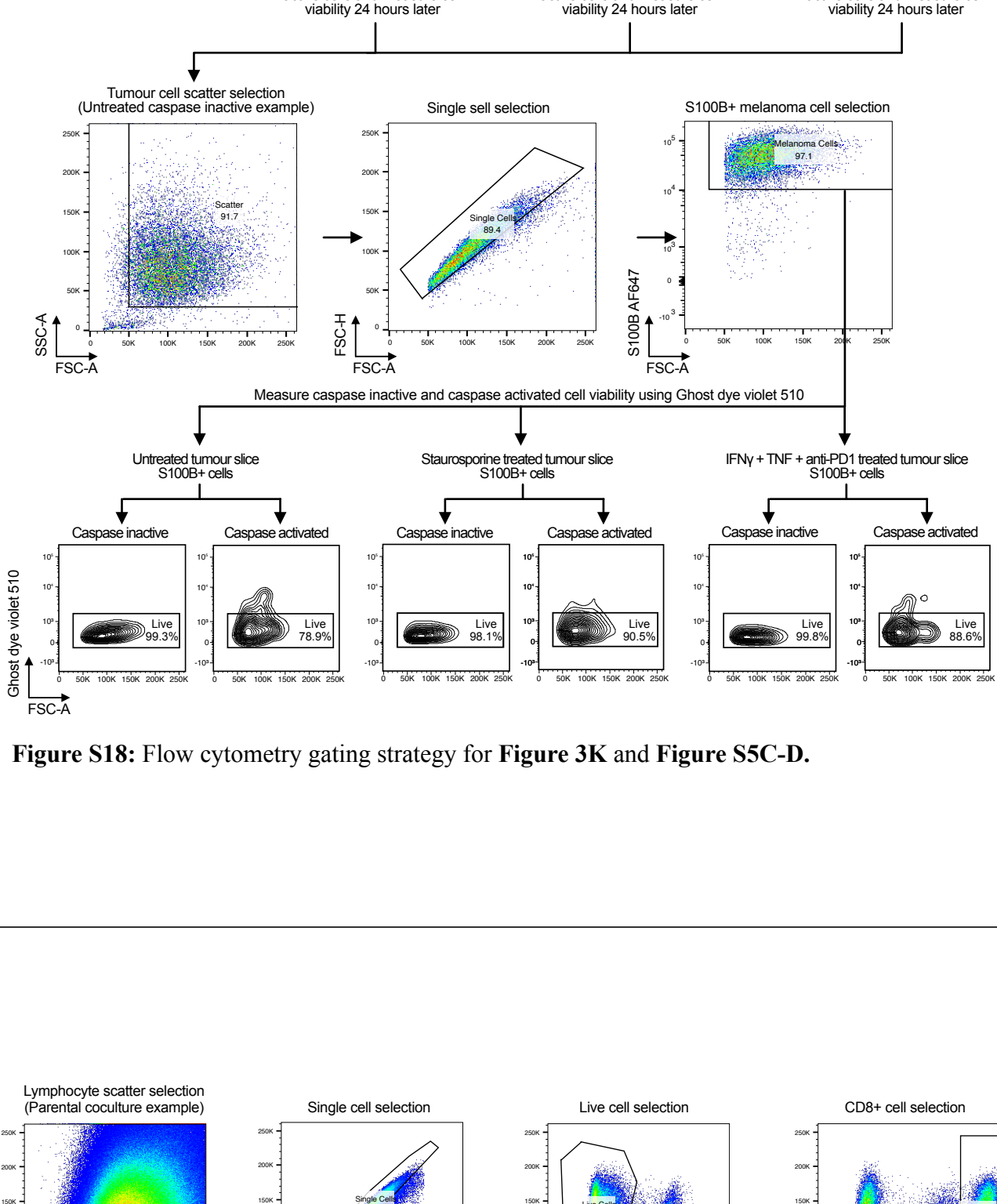

Supplement: Supplement 3 — Document S3. Figures S11–S19 (gating strategies for flow cytometry experiments) [file media-3.pdf]
